# Regulation of miR-1-Mediated Connexin 43 Expression and Cell Proliferation in Dental Epithelial Cells

**DOI:** 10.3389/fcell.2020.00156

**Published:** 2020-03-17

**Authors:** Tomoaki Nakamura, Tsutomu Iwamoto, Hannah M. Nakamura, Yuki Shindo, Kan Saito, Aya Yamada, Yoshihiko Yamada, Satoshi Fukumoto, Takashi Nakamura

**Affiliations:** ^1^Division of Pediatric Dentistry, Department of Oral Health and Development Sciences, Tohoku University Graduate School of Dentistry, Sendai, Japan; ^2^Department of Pediatric Dentistry, Institute of Biomedical Sciences, Tokushima University Graduate School, Tokushima, Japan; ^3^Division of Nephrology and Endocrinology, Tohoku Medical and Pharmaceutical University, Sendai, Japan; ^4^Division of Molecular Pharmacology and Cell Biophysics, Department of Oral Biology, Tohoku University Graduate School of Dentistry, Sendai, Japan; ^5^Laboratory of Cell and Developmental Biology, National Institute of Dental and Craniofacial Research, NIH, Bethesda, MD, United States

**Keywords:** dental development, differentiation, microRNA, cell proliferation, connexin 43

## Abstract

Many genes encoding growth factors, receptors, and transcription factors are induced by the epithelial-mesenchymal interaction during tooth development. Recently, numerous functions of microRNAs (miRNAs) are reportedly involved in organogenesis and disease. miRNAs regulate gene expression by inhibiting translation and destabilizing mRNAs. However, the expression and function of miRNAs in tooth development remain poorly understood. This study aimed to analyze the expression of miRNAs produced during tooth development using a microarray system to clarify the role of miRNAs in dental development. miR-1 showed a unique expression pattern in the developing tooth. miR-1 expression in the tooth germ peaked on embryonic day 16.5, decreasing gradually on postnatal days 1 and 3. An *in situ* hybridization assay revealed that miR-1 is expressed at the cervical loop of the dental epithelium. The expression of miR-1 and connexin (Cx) 43, a target of miR-1, were inversely correlated both *in vitro* and *in vivo*. Knockdown of miR-1 induced the expression of Cx43 in dental epithelial cells. Interestingly, cells with miR-1 downregulation proliferated slower than the control cells. Immunocytochemistry revealed that Cx43 in cells with miR-1 knockdown formed both cell-cell gap junctions and hemichannels at the plasma membrane. Furthermore, the rate of ATP release was higher in cells with miR-1 knockdown than in control cells. Furthermore, Cx43 downregulation in developing molars was observed in Epiprofin-knockout mice, along with the induction of miR-1 expression. These results suggest that the expression pattern of Cx43 is modulated by miR-1 to control cell proliferation activity during dental epithelial cell differentiation.

## Introduction

Tooth development is regulated by a series of epithelial-mesenchymal tissue interactions to form morphologically optimized functional cusps. Tooth development can be divided into four stages: initiation [embryonic day (E) 11.5], bud (E13.5), cap (E15.5), and bell (E17.5) (Nakamura et al., 2004). Studies using knockout and transgenic mice have elucidated the complex genetic regulation of tooth development and growth to control the crown of teeth. However, each tooth shows morphologically different shape as well as size oral cavity in humans. To fabricate these varied tooth shapes, the regulatory mechanism of each gene involved in tooth development may be different.

microRNAs (miRNAs or miRs) are small non-coding RNAs (18–22 nt) involved in regulating post-transcriptional gene expression by controlling the stability and translation of mRNAs in organogenesis and diseases (Gregory et al., 2006).

Although millions of promoter activity regions have been identified in the human genome via FANTOM CAGE analysis at Riken, the mechanisms underlying transcriptional regulation in individual non-coding RNAs, small RNAs, and miRNAs remain unknown (Brennecke et al., 2005; Kawaji et al., 2011). *In silico* analysis predicted that conserved vertebrate miRNAs target more than 400 regulatory genes (Bartel, 2004, 2009). Diverse miRNA functions have been reported in essential cellular phenomena including cell proliferation, differentiation, and cell-type specification in studies on dicer-null mice. Dicer is required for the processing of most miRNAs and for digesting long dsRNAs into small interfering RNAs (Bernstein et al., 2003; Kloosterman and Plasterk, 2006).

The dental phenotypes of epithelial-specific conditional knockout dicer mice using cytokeratin 14-Cre (*Dcr^*K*14–/–^*) and Pitx2-Cre (*Dcr^*Pitx*2–/–^*) have been reported (Cao et al., 2010; Michon et al., 2010). Germline pathogenic variation in DICER1 causes dental abnormalities. Conditional inhibition of miRNA production in the epithelium of the tooth germ reportedly resulted in significant aberrations in molar cusp patterning and grooves on the labial surface of the incisors. Furthermore, *Dcr^*K*14–/–^* mice displayed impaired dental epithelial cell differentiation into ameloblasts and deficient enamel formation both in molars and incisors. *Dcr^*Pitx*2–/–^* mice had relatively more severe phenotypes than *Dcr^*K*14–/–^* mice. In *Dcr^*K*14–/–^* and *Dcr^*Pitx*2–/–^* mice, the entire miRNA production was blocked in epithelial cells; hence, limited information is available regarding the expression and roles of miRNA in tooth development.

One of the miR-1 target genes is *Gja-1* (gap junction protein, alpha-1) which encodes connexin 43 (Cx43) gap junction proteins (Yang et al., 2007; Xu et al., 2012). Cx43 is expressed on the plasma membrane of cells and forms a connexon: a protein complex comprising six connexin proteins. The connexon structure is essential for the functioning of gap junctions. Cx43 was initially identified as a tumor suppressor gene owing to an inverse correlation between tumor malignancy and Cx43 expression in tumor cells (Plante et al., 2011). Although the mechanism through which Cx43 inhibits cell proliferation remains unknown, the connexin hemi-channel potentially contributes to intracellular ATP release to the extracellular milieu (Batra et al., 2012). Depletion of intracellular ATP potentially suppresses cell growth (Cheng et al., 2014; Chi et al., 2014).

Oculodentodigital dysplasia (ODDD) is an autosomal dominant human disease caused by mutations in *GJA-1*, which encodes Cx43. ODDD syndrome is characterized by small eyes, abnormal face shape, syndactyly of the fourth and fifth fingers and toes, and severe hypoplastic enamel (van Es et al., 2007; Porntaveetus et al., 2017).

Epiprofin (Epfn) is a master gene in ameloblast differentiation, belonging to the Sp family of transcription factors (Nakamura et al., 2011; Aurrekoetxea et al., 2016). Epfn is expressed in developing teeth, hair follicles, skins, limb, and genitals. Epfn-deficient mice have supernumerary tooth formation, enamel hypoplasia, abnormal hair follicle formation, skin abnormality, and oligodactyly with a distal bifurcation of synostotic digits and cutaneous syndactyly (Nakamura et al., 2008, 2014; Talamillo et al., 2010). The expression patterns of Epfn and *Gja-1* are overlapped especially in developing teeth and limbs (Richardson et al., 2004; Nakamura et al., 2008; Talamillo et al., 2010). During limb and tooth development, *Epfn*-deficient mice develop similar phenotypes to those of *Gja-1* null mice and are used as animal models of ODDD syndrome (Richardson et al., 2004). However, ODDD patients do not present with supernumerary teeth, which is observed in Epfn-deficient mice.

A better understanding of the role of miRNAs in tooth development would elucidate their role in prominent diseases including ODDD and further the understanding of this complex developmental process. Herein, we analyzed the expression profiles of miRNAs during tooth development, particularly focusing on miR-1. We used knockdown miR-1 cells and molecular methods to elucidate the association between miR-1 expression and Cx43 at various stages of tooth development.

## Materials and Methods

### Cell Culture and Transfection of the miR-1 Knockdown Probe

The rat-derived dental epithelial cell line, SF2, was cultured at 37°C under 5% CO_2_ in Ham F-12/Dulbecco’s modified Eagle’s medium supplemented with 10% fetal bovine serum (Nakamura et al., 2017). To knockdown miR-1 in SF2 cells, we used LNA miR-1 knockdown probes labeled with FITC or non-labeled probes (Exiqon, Denmark), with five nucleotides or deoxynucleotides at both ends of the antisense molecule locked (LNA; the ribose ring is constrained by a methylene bridge between the 2′-*O*- and the 4′-*C*atoms) (Jorgensen et al., 2010). The sequence of LNA-antimiR-1 was 5′-ACTTCTTTACATTCC-3′. A scrambled sequence was used as a negative control: 5′-ATCTTACTTATCCTC-3′. miR-1 knockdown or scramble control probes (5′-ACGTCTATACGCCCA-3′) were transfected using lipofectamine 2000 and PLUS reagent (Thermo Fisher Scientific, CA, United States).

### miRNA Extraction and miRNA Array Analysis

Small RNAs were purified from ICR mice developing molars on E16.5, post-natal 1 day (P1), and P3 using the mirVana miRNA isolation kit^TM^ system (Ambion, TX, United States) in accordance with the manufacturer’s instructions. To identify the differentially expressed miRNAs in the developing tooth, we utilized the Genopal^TM^ miRNA gene chip system. Preparation of small-sized RNA, hybridization, and signal detection were performed in accordance with the Genopal protocol (Mitsubishi Rayon, Japan) (Jorgensen et al., 2010). The raw data will be made available without undue reservation to any qualified researcher. The miRNA expression data for this study are available in the Gene Expression Omnibus (GEO) under accession number GSE141608.

### Cell Proliferation Assay

Cell proliferation was measured using a WST assay (Dojindo, Japan) and via incorporation of 5-bromo-2′-deoxyuridine (BrdU). Four-thousand SF2 cells incubated with Anti-BrdU antibodies were visualized using an Alexa 594-conjugated secondary antibody (Thermo Fisher Scientific) (Nakamura et al., 2016). Nuclei were stained with Hoechst 33342 dye (Thermo Fisher Scientific). A BrdU incorporation assay was performed using 5-bromo-2′-deoxyuridine labeling and a detection kit (Sigma-Aldrich, MO, United States) in accordance with the manufacturer’s instructions (Ibarretxe et al., 2012). SF2 cells transfected with either knockdown miR-1 or scramble probes were cultured on a glass slide for 24 h. Before fixation with glycine/methanol, cells were labeled with BrdU for 1 h. BrdU was detected in accordance with the manufacturer’s protocol.

### *In situ* Hybridization, Immunohistochemistry, and Immunocytochemistry

FITC-labeled single-strand locked nucleic acid (LNA) RNA probes for miR-1 and U6 were obtained from Exiqon (Qiagen, Germany). LNA probes were hybridized in accordance with the manufacturer’s instructions. Frozen tissue sections were obtained from heterozygous or homozygous Epfn-deficient-mouse heads (E16.5, P1, and P3) containing molars, and were placed on RNase-free glass slides (Nakamura et al., 2008). The SF2 cells on glass slides were transfected with either miR-1 knockdown or scramble probes and cultured for 48 h and fixed with 4% paraformaldehyde in PBS for 5 min. Primary anti-connexin 43 (1:400 dilution, Santacruz Biotechnology, CA, United States), anti-*E*-cadherin (1:200 dilution, BD Pharmingen, CA, United States), and anti-Epiprofin antibodies (1:400 dilution) were visualized using Alexa 488 or Alexa 594-conjugated secondary antibodies (1:500 dilution, Thermo Fisher Scientific) (Nakamura et al., 2008). Nuclear staining was performed with Hoechst dye (Thermo Fisher Scientific). Immunohistochemistry, *in situ* hybridization, or immunocytochemistry were performed independently in triplicate. Images for immunohistochemistry, *in situ* hybridization, and immunocytochemistry were captured using a BZ-8000 microscope (KEYENCE, Japan). Histological analysis was performed using BZ analyzer (KEYENCE). The experimental animal protocol for maintaining mice was approved by the Institutional Animal Care Committee of Tohoku University (No. 2017DnA-045).

### Western Blotting

SF2 cells transfected with either knockdown miR-1 or scramble probes were lysed in M-per buffer plus protease inhibitor cocktail (Sigma-Aldrich). The samples were centrifuged (14000 rpm, 5 min, 4°C), and the supernatants were diluted in NuPage LDS buffer and then separated on a 4–20% NuPage Bis-tris gradient gel (Thermo Fisher Scientific). Separated proteins were electro-transferred on to PVDF membranes (Thermo Fisher Scientific). After blocking with 3% non-fat skim milk in PBS, the membrane was probed with anti-Cx43 (Abcam, United Kingdom) or anti-β-actin primary antibodies (1:100 dilution, Abcam) in PBS. The primary antibodies were detected using HRP-conjugated anti-rabbit IgG secondary antibodies (1:500 dilution, Thermo Fisher Scientific) using an ECL kit (Amersham Biosciences Co., NJ, United States) and a LAS 4000 UV mini system (Fujifilm-GE healthcare, Japan).

### Real-Time RT-PCR Analysis

Small RNAs were purified from homogenized mouse tissue (ICR, Epfn^±^, Epfn^–/–^) of developing molars on E16.5, P1, and P3, using a miRVanaTM miRNA isolation kit (Thermo Fisher Scientific) and a Micro Smash^TM^ (TOMY, Japan) in accordance with the manufacturer’s instructions. miRNA cDNA was synthesized using the TaqMan^TM^ miRNA Reverse Transcription Kit (Thermo Fisher Scientific) with mmu-miR-1. Real-time PCR was performed using a standard TaqMan^TM^ PCR protocol on an Applied Biosystems StepOne^TM^ real-time PCR System (Thermo Fisher Scientific). The reaction protocol was as follows: 95°C for 10 min, followed by 40 cycles at 95°C for 15 s and 60°C for 1 min.

Total RNA was extracted from mouse molars in Epfn± or −/− mice using the ISOGEN II reagent (Nippon gene, Japan). After 2 U of DNaseI (Sigma-Aldrich) treatment, 1 μg of total RNA was reverse-transcribed using SuperScript^®^ VILO^TM^ Master Mix (Thermo Fisher Scientific) to generate cDNA, which was used as a template for PCR reactions with gene-specific primers (Table 1). For semiquantitative RT-PCR, cDNA was amplified with an initial denaturation step of 95°C for 3 min, then 95°C for 30 s, 60°C for 30 s, and 72°C for 30 s for 30 cycles, and a final elongation step at 72°C for 5 min. The RT-PCR products were separated on a 2% (w/v) agarose gel in Tris-acetate EDTA buffer, stained with SYBR^TM^ safe DNA Gel Stain (Thermo Fisher Scientific), and viewed under UV light, using a LAS 4000 UV mini system (Fujifilm-GE healthcare).

**TABLE 1:** Primer sequence using RT-PCR analysis.

**Gene**	**Sequence**
mEpiprofin	5′-TCTCACTATTTCACCCTCCCCTG-3′
	5′-ACCTCATCTCTGCTTTCTCTCCG-3′
mGja-1	5′-TTGGGGGGTGTTTTGGGATAGC-3′
	5′-TTAGCGGGGATGTAGGACAACCTG-3′
mHPRT	5′-GCGTCGTGATTAGCGATGATGA-3′
	5′-GTCAAGGGCATATCCAACAACA-3′

### Quantification of ATP Release in miR-1 Knockdown SF2 Cells

The amount of ATP released was determined via luminometry, using an ATP detection kit (Promega, WI, United States) in accordance with the manufacturer’s instructions. Briefly, SF2 cells transfected with either knockdown miR-1 or scramble probes were seeded at 1 × 10^3^ cells/well in a 96-well plate and incubated for 24 h. The supernatant was harvested and assayed with luciferase/luciferin. Released ATP was relatively quantified by detecting the fluorescence signal emitted in the luciferase-mediated reaction of D-luciferin with ATP into D-oxyluciferin and measured using a GloMax^®^ 20/20 Luminometer (Turner BioSystems, CA, United States).

### Statistical Analysis

Each experiment was performed independently in triplicate. Data are presented as mean ± standard deviation (SD) values. The Student’s *t*-test and the one-way analysis of variance (ANOVA) were used. When the standard deviations were significantly different between groups, the Kruskal–Wallis non-parametric ANOVA test were used. A *P*-value of <0.05 was considered significant. Data analysis was performed using Prism8^TM^ software (GraphPad Software, CA, United States).

## Results

### miRNA Expression Profiling During Tooth Development

Because the shape of the crown is determined after the cap stage, and dynamic cytodifferentiation occurs after the bell stage, we selected the characteristic stages of molar development at E16.5, P1, and P3. We prepared total RNA from tooth germ at E16 and newborn stages plus postnatal (P) 3 days, representing mature tooth development, for microarray-based comparison. The miRNA microarray data of these three stages of tooth development were compared (Supplementary Figure S1).

**FIGURE 1:**
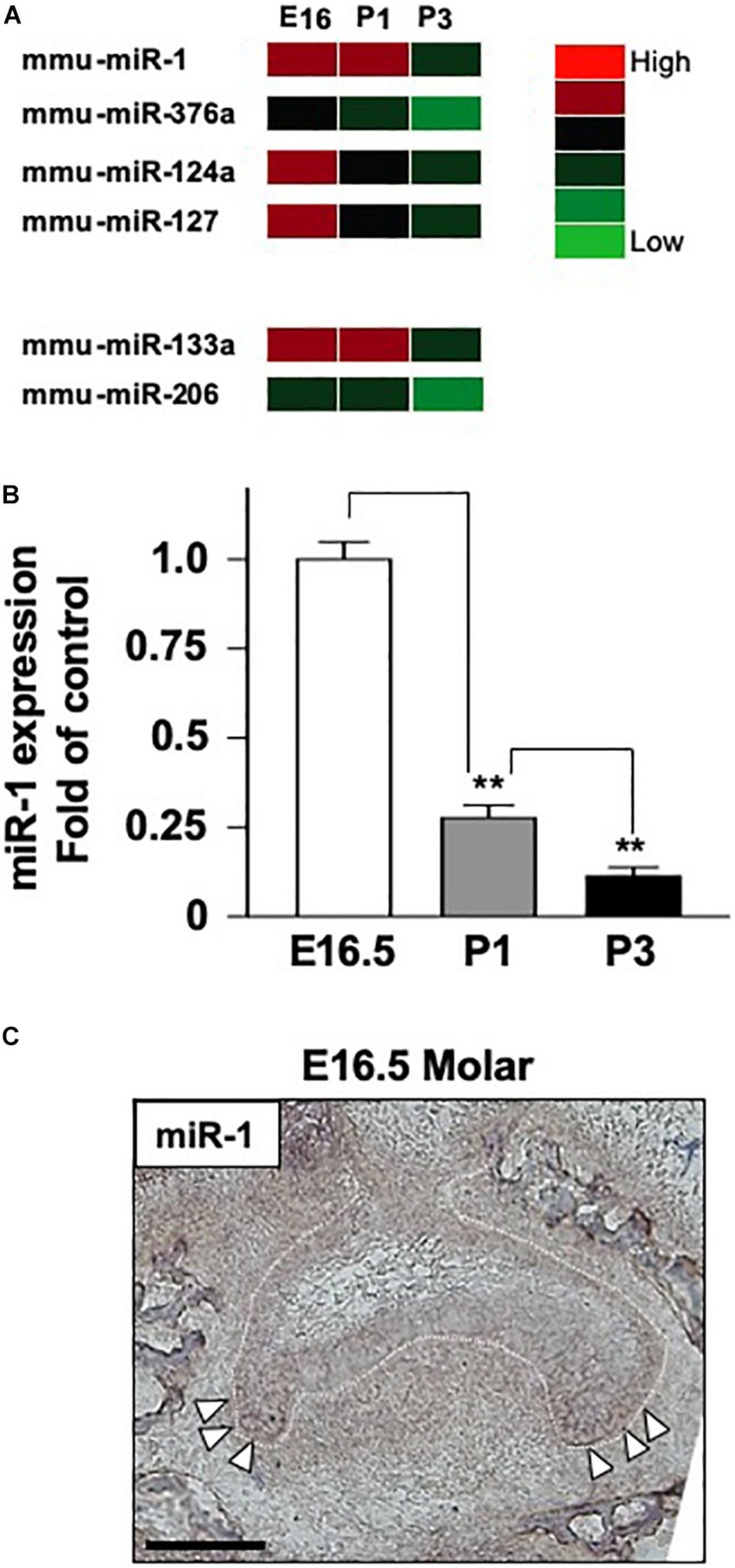
The expression of miR-1 during tooth development. **(A)** miRNA microarray data at three stages (E16.5, P1, P3) during tooth development. High expression is indicated by red-colored bars, and low expression is indicated by light green colored bars. Heat map analysis of miRNA-1, -376a, -124a, and -127, which were down-regulated with tooth developmental stages. **(B)** Real-time PCR analysis of miR-1 expression at E16.5 and P3. miR-1 was highly expressed at E16.5 in the tooth germ but had significantly lower expression at P3 than E16.5. Data are presented as mean ± standard deviation (SD) values. The statistical analysis was done by the Kruskal–Wallis non-parametric ANOVA test (**P < 0.01). **(C)** miR-1 was detected at both sides of the cervical loop in the E16.5 tooth bud (white arrowheads). The white dot line indicates the margin of the dental epithelium and the mesenchyme. *n* = 3, Scale bar 100 mm.

Fifty-seven miRNAs were significantly upregulated or downregulated during tooth development. Interestingly, miR-1, miR-376a, miR-124a, and miR-127 displayed particularly dynamic expression changes during tooth development (Figure 1A). miR-1 was strongly expressed at E16 and P1 but drastically reduced at P3 (Figure 1A). As expected, the expression profiles of miR-133a and miR-206, which are cluster miRNAs with miR-1, were synchronized with that of miR-1 (Figure 1A).

### TaqMan^TM^ Analysis of miR-1 Expression During Tooth Development

In the heatmap generated on the basis of the miRNA microarray during tooth development, miR-1 was upregulated on E16.5 and was significantly downregulated on P3. Therefore, we validated miR-1 expression in the tooth germ at these stages using the TaqMan^TM^ system. As expected, miR-1 expression was detected using TaqMan^TM^ primer and probe system in E16 tooth germ cDNA (Figure 1B). We observed a 90% reduction in miR-1 expression in the P3 tooth germ compared to in the E16.5 tooth germ (Figure 1B). These results validate the present microarray results and confirm the unusual expression pattern of miR-1 during tooth development.

### The Tissue-Specific Expression Pattern of miR-1 During Tooth Development

We confirmed that the miR-1 expression was significantly altered during tooth development in homogenized tissue; hence, we performed tissue-specific *in situ* hybridization of miR-1 to identify the spatial pattern of expression during tooth development. miR-1 in the developing molar on E16.5 was primarily localized in the cervical loop, which contains highly proliferating dental epithelial cells (Figure 1C), suggesting that miR-1 potentially regulates cell proliferation in dental epithelial cells.

### Negative Regulation of Cx43 Expression by miR-1 in Dental Epithelial Cells

**FIGURE 2:**
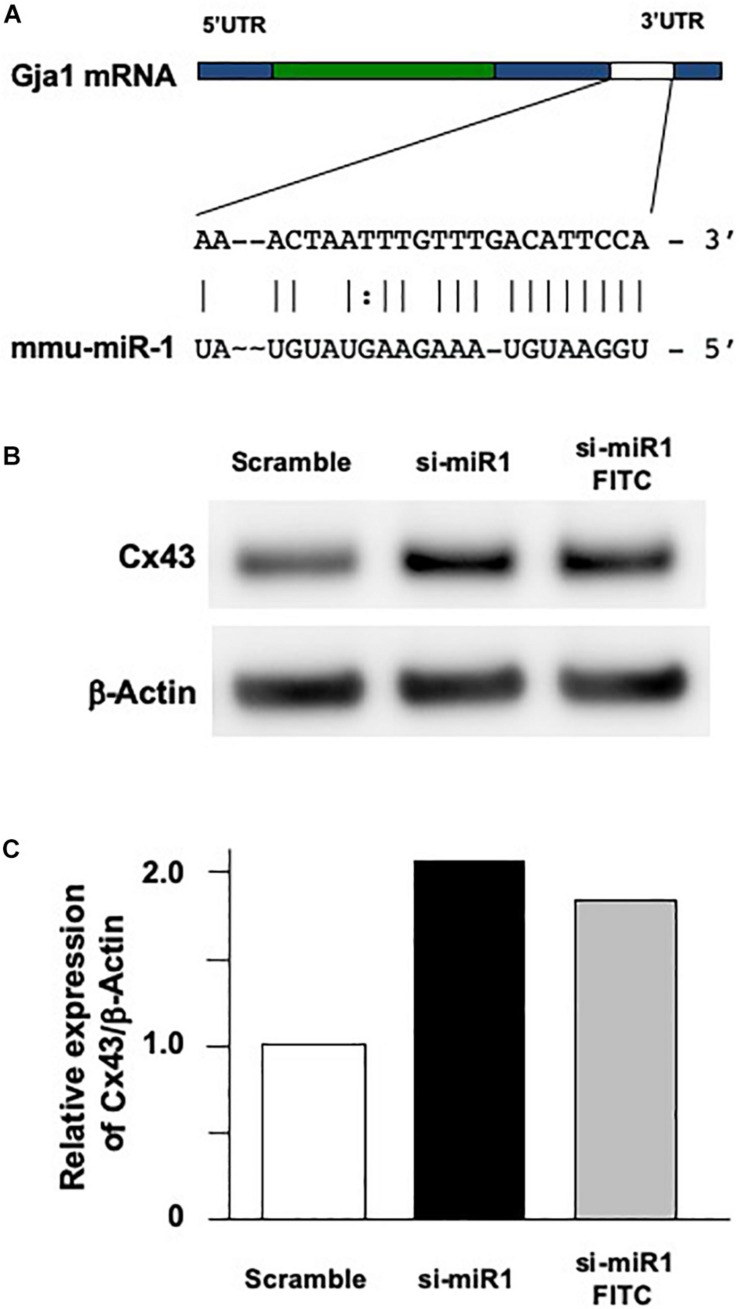
Effect of Cx43 expression following miR-1 knockdown using siRNA (si-miR-1) in dental epithelial cells. **(A)** Gene structure of mouse *Gja-1*. The untranslated regions (UTRs) located at the 5′ and 3′ ends are indicated with blue boxes, and the open reading frame of *Gja-1* is indicated with the green box. The miR-1 target region in the 3′ UTR is indicated with a white box. Schema of the potential binding site for miR-1 (mmu-miR-1) mature sequences in the 3′ UTR of gla-1, which encodes Cx43. The complementarity sequences are connected by a vertical line, and the G:U/U:G wobble is indicated by bold letters connected by dots. **(B)** Western blot analysis for Cx43 in SF2 using antibodies against Cx43. The knockdown of miR-1 using a miR-1 knockdown probe promoted the expression of Cx43 protein in SF2 cells compared to in cells transfected with a scrambled probe used as the control. **(C)** Quantification of the expression of Cx43 normalized to the expression of β-actin in SF2 co-transfected with either the miR-1 knockdown or the scramble probe.

One of the known targets of miR-1 is Gja-1, which encodes the Cx43 gap junction protein (Figure 2A). miR-1 interferes with the translation of *Gja-1* into Cx43 in numerous cell types. Hence, we investigated the effect of miR-1 on Cx43 production, using a dental epithelial cell line, SF2, using a miR-1 knockdown system. The efficiency of incorporation of small interfering miR-1 (si-miR-1) and control (scramble) siRNA was approximately 70% of the total cells (Supplementary Figure S2). Cx43 expression in the control (Scramble in Figure 2B) was detected as a single band at the expected size. The miR-1 knockdown cells, either by si-miR1 or si-miR1-FITC conjugated probe, expressed Cx43 at a higher level than that of the control (Figure 2B). Band intensities (Figure 2B) were quantified and normalized to the expression levels of the internal control β-actin. The expression level of Cx43 in miR-1 knockdown cells, by either si-miR1 or si-miR1-FITC conjugated probe, increased by twofold compared to that of the control (scramble transfected cells) (Figure 2C).

### The Role of miR-1 in Dental Epithelial Proliferation

**FIGURE 3:**
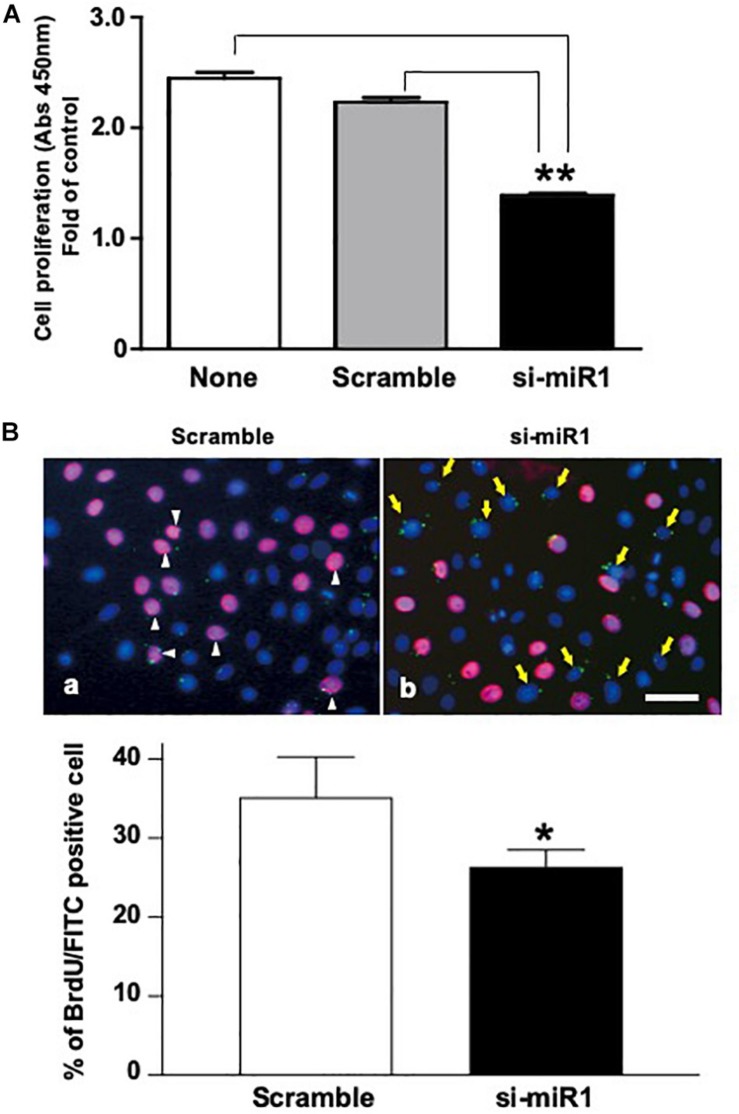
Promotion of cell proliferation in miR-1-knockdown SF2 cells using si-miR1 probes. **(A)** Cell proliferation assay. SF cells transfected with miR-1 knockdown had reduced cell proliferation compared to cells transfected with scramble, or no probe (denoted ‘none’). Data are presented as mean ± standard deviation (SD) values. The statistical analysis was done by the Kruskal–Wallis non-parametric ANOVA test (**P < 0.01) **(B)** BrdU incorporation assay with FITC labeled miR-1 or scramble probes. The cell nuclei that had incorporated BrdU were stained red, while miR-1 knockdown (si-miR1) (yellow arrows in **b**) or scramble (white arrowheads in **a**) probe-transfected cells were stained green with FITC. Nuclear staining was performed with Hoechst 33342 (blue). Scale bar 100 mm There was a 10% reduction in the amount of BrdU incorporation in SF cells that were co-transfected with si-miR1-FITC compared to that of scramble-FITC. Data are presented as mean ± standard deviation (SD) values. The statistical analysis was done by the Student’s *t*-test (*P < 0.05).

To investigate the cellular functions of miR-1, we measured cell proliferation after blocking miR-1 expression with si-miR1. We observed a reduction in cell proliferation activity via the WST-8 assay in miR-1 knockdown cells compared to non-treated siRNA or scramble control oligo-transfected cells (Figure 3A). miR-1 thus positively regulates proliferation in dental epithelial cells. To confirm these results, we also assessed cell proliferation using a BrdU incorporation assay. si-miR1 labeled with FITC, visualized as a green signal, incorporated less BrdU (red) than the control (Figure 3B, arrows). To quantify these results, we enumerated the BrdU/FITC double-positive cells and probe-incorporating FITC-positive cells. Approximately 35% of scramble FITC positive cells were positive for BrdU (red) (Figure 3B, arrowheads). However, approximately 25% of cells with miR-1 knockdown-FITC probes also incorporated BrdU (red), indicating a 10% reduction in the number of BrdU/FITC-double-positive cells upon miR-1 knockdown (Figure 3B). Furthermore, no significant differences were observed in nuclear morphology between scramble and si-miR-1-transfected cells, suggesting that miR-1 knockdown diminished cell proliferation without inducing cell death (Figure 3B). Based on these results, the expression of miR-1 appears to be required for proper cell proliferation during tooth development.

### The Regulation of Cx43 Cellular Localization by miR-1 in Dental Epithelial Cells

**FIGURE 4:**
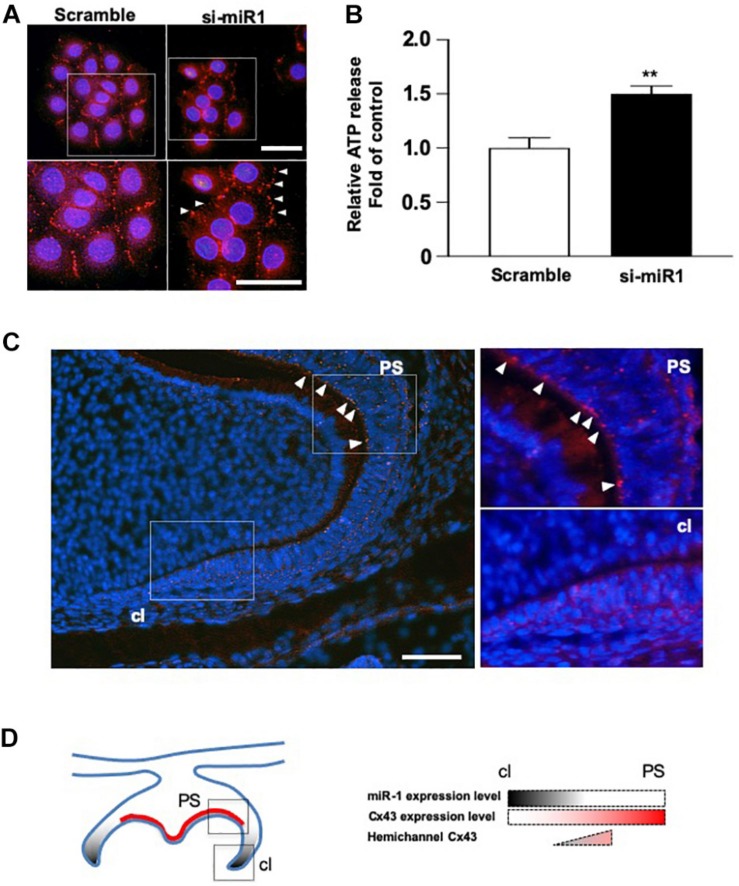
Dynamic changes in the cellular localization of Cx43 following miR-1 knockdown. **(A)** Immunocytochemistry analysis of Cx43 in control (Scramble) and miR-1 knockdown (si-miR1) dental epithelial cells. Higher magnification images are shown in the boxed area below. Arrowheads indicate hemichannel formation by Cx43 following the knockdown of miR-1. Scale bar 50 mm. **(B)** Effect of miR-1 on the release of extracellular ATP in control dental epithelial cells (Scramble) and miR-1 knockdown cells (si-miR1). Data are presented as mean ± standard deviation (SD) values. The statistical analysis was done by the Student’s *t*-test (**P < 0.01). **(C)** The expression pattern of hemichannel Cx43 in developing molars (P1). Differentiating presecretory ameloblasts express hemichannel Cx43 (arrowheads). Gap junctions were formed in dental epithelial cells at the cervical region. Scale bar 100 mm. **(D)** Schematic representation of miR-1 and Cx43 localization in the developing molar. An inverse gradient of expression was observed between miR-1 and Cx43, as well as in the distribution of hemichannel Cx43 in the developing molar. PS, presecretory ameloblast; cl, cervical loop.

Cx43 cellular localization was analyzed in SF2 cells after miR-1 knockdown. While in scramble (control) cells, Cx43 was expressed on the plasma membrane at the cell–cell junction, indicating that Cx43 forms part of the gap junction. In miR-1 knockdown SF2 cells, Cx43 was localized on the plasma membrane at the sides that were not adjacent to other cells and at the cell-cell junction (Figure 4A), suggesting that excess Cx43 induced by knockdown of miR-1 also accumulates at the hemichannels. Cultured astrocytes are known to release glutamate and ATP in divalent cation-free media via gap junction hemichannels (Stout et al., 2002). Hence, we quantified ATP release from dental epithelial cells transfected with si-miR1 or scramble control probes. The amount of extracellular release of ATP from miR-1 knockdown dental epithelial cells was significantly higher than that in control cells transfected with the scramble probe (Figure 4B). miR-1 may therefore regulate cell proliferation by regulating Cx43 production and Cx43 hemichannel formation. To verify our *in vitro* analysis, we observed hemichannel Cx43 localization in developing molars. Cx43 was expressed in both dental epithelial cells and presecretory ameloblasts, which are post-mitotic cells. Cx43 localized on the cellular plasma membrane adjacent to other cells, indicating that Cx43 forms gap junctions (Figure 4C). Interestingly, Cx43 was downregulated in dental epithelial cells at the cervical region, where miR-1 is expressed, compared with the inner dental epithelium (Figure 1C), suggesting that miR-1 localized expression may inhibit Cx43 expression. Cx43 was also detected at the sides of the basal membrane in addition to the cell-cell contact plasma membrane (Figure 4C, arrowheads). Cx43 localized at the sides of the basal membrane in presecretory ameloblasts did not form gap junctions, which are expressed by other cells (Figure 4C). These results suggest that Cx43 expressed by presecretory ameloblasts forms both gap junctions and hemichannels, and the formation of hemichannels may inhibit cell proliferation during ameloblast differentiation, as shown in Figure 4D.

### The Induction of miR-1 Expression in Molars of Epfn-Deficient Mice

**FIGURE 5:**
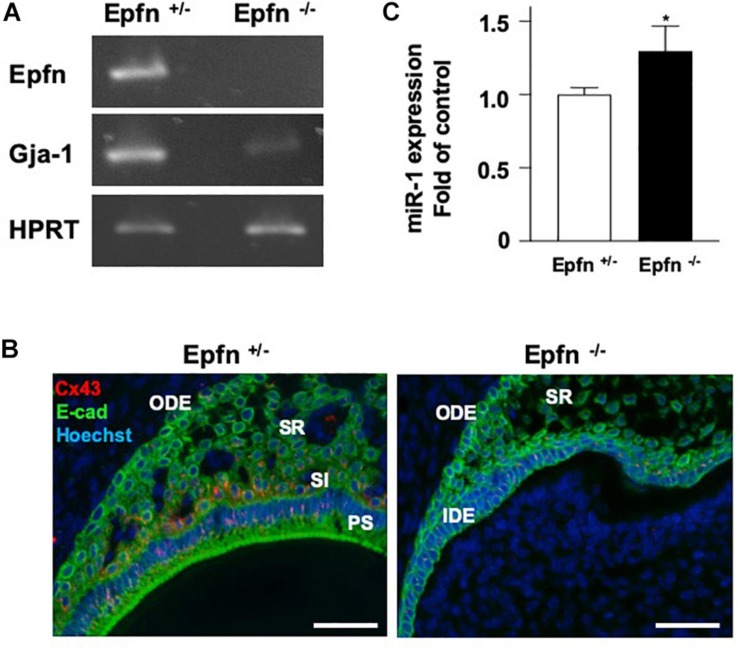
Reduced expression of Cx43 in Epfn-deficient molars. **(A)** RT-PCR comparison of *Epfn* and *Gja-1* gene expression in the P1 molars of Epfn^±^ or Epfn^–/–^ mice. HPRT was used as an internal control. *P < 0.05 **(B)** Immunocytochemistry analysis of Cx43 (red) and *E*-cadherin (green) in dental epithelial cells of P1 Epfn heterozygous (Epfn^±^) or Epfn-deficient (Epfn^–/–^) mouse molars. The nucleus was stained with Hoechst 33342 (blue). PS, presecretory ameloblast; SR, stellate reticulum; SI, stratum intermedium; IDE, inner dental epithelium; ODE, Outer dental epithelium, scale bar 50 mm. **(C)** Real-time PCR analysis of miR-1 expression in P1 molars of Epfn^±^ or Epfn^–/–^ mice. Statistical comparisons were performed using the Student’s *t*-test, *P < 0.05.

The expression of *Gja-1*, encoding Cx43, was greatly reduced in Epfn^–/–^ molars (Figure 5A). Immunohistochemical analysis with an anti-Cx43 antibody revealed reduced expression of Cx43 in Epfn^–/–^ mouse molars compared to in Epfn^±^ mice (Figure 5B). Because Epfn is a master gene in ameloblast differentiation, dental epithelial cell differentiation is completely blocked Epfn^–/–^ mouse molars. Next, we measured the expression of miR-1 in undifferentiated dental epithelial cells in Epfn^–/–^ mouse molars using TaqMan^TM^ and observed increased miR-1 expression in Epfn^–/–^ molars compared to Epfn^±^ molars (Figure 5C). These results support the inverse correlation of the expression between miR-1 and Cx43 observed in developing molars, and suggest that the attenuation of miR-1 is important for the differentiation of dental epithelial cells into ameloblasts.

## Discussion

**FIGURE 6:**
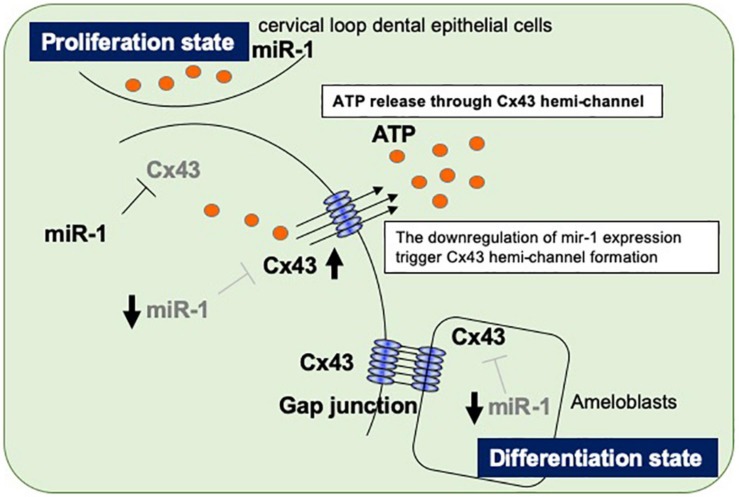
Proposed model of the regulatory mechanism in ameloblasts by miR-1-mediated Cx43 expression during dental epithelial cell differentiation. Dental epithelial cells at the cervical loop area proliferate rapidly and express miR-1, which block Cx43 expression. Proliferating dental epithelial cells start to express Cx43 by the attenuation of miR-1 expression and form hemichannel Cx43 to release intracellular ATP. The inverse expression between miR-1 and Cx43 is observed during dental epithelial cell differentiation into ameloblasts.

The regulation of organogenesis by miRNAs has been studied using dicer-conditional KO mice cross-mated with specific interest Cre transgenic mice. Cytokeratin 14-Cre and Pitx2-Cre mice have been used to generate dicer knockdown mice in epithelial cell lineages, while Wnt1-Cre mice have been used to generate dicer knockdown mice in neural crest-derived mesenchyme lines (Cao et al., 2010; Michon et al., 2010). *Dcr^*K*14–/–^* mice display impaired ameloblast differentiation and enamel formation with abnormal crown shapes (Michon et al., 2010), and the dental epithelium in *Dcr^*Pitx*2–/–^* mice forms extra cell niches, which develop extra incisors (Cao et al., 2010). *Dcr^*Wnt*1/–^* mice, which have defective miRNA production in neural crest derived-cell lineages, have extremely severe craniofacial abnormalities and missing tooth buds (Cao et al., 2010). These reports imply that miRNAs play essential roles in normal tooth development. To date, there have been reports of many different miRNAs expressed during tooth development, both in the dental epithelium and in the mesenchyme, but the function of specific miRNAs remains unclear. In the present study, we performed high-throughput analysis of miRNAs expressed during tooth development and focused on characterizing the role of miR-1 (Figure 6). Connexin 43 (Cx43), a gap junction protein, is a target molecule of miR-1, and no connexins other than Cx43 have been identified as a target gene of miR-1 (Klotz, 2012). Our results suggest that *Gja-1* is a target gene of miR-1 in dental epithelial cells, as in cardiomyocytes (Yang et al., 2007). Although connexins including Cx26, Cx32, and Cx43 are expressed in dental epithelial cells, Cx43 knockdown in developing teeth causes a severe enamel hypoplasia, suggesting that Cx26 and Cx32 cannot functionally compensate for Cx43 (Toth et al., 2010). Cx43 is most predominantly expressed during development especially in dental epithelial cells; however, it is downregulated in mature adult teeth but expressed in carious teeth localized at odontoblast processes owing to the obliteration of ameloblasts after the enamel formation in developing teeth (About et al., 2002; Ibuki et al., 2002). Gap junctions are made of two hemichannels from two neighboring cells across the extracellular gap. Each hemichannel, or connexon, is constructed from six connexin proteins. Standalone hemichannels can also be present in cells. These hemichannels have a low opening probability that is increased under various physiological and pathological conditions (Saez et al., 2005; Spray et al., 2006). We hypothesize that, besides mediating cell-cell communication, undocked connexin channels allow communication between the intracellular and extracellular milieu, thereby playing an important role in paracrine communication during tooth development. Recently we reported that pannexin 3 (Panx3), a hemi-channel protein, is also preferentially expressed in the tooth germ, controls cell proliferation and differentiation in dental mesenchymal cells, which differentiate into dentin-forming odontoblasts (Iwamoto et al., 2017). Panx3 hemichannels are locally expressed in pre-odontoblasts. Panx3 blocks cell proliferation and promotes differentiation into odontoblasts in pre-odontoblasts by releasing intracellular ATP to the extracellular space. The tooth is covered with epithelial-derived enamel and mesenchyme-derived dentin to confer physical hardness. These two hard tissues are distinctly built up in daily cycles by the layer structures to accumulate enamel- or dentin-matrices (Kawasaki et al., 1979). The regulation of dental cell differentiation should be controlled simultaneously to create the enamel or dentin surface. Although there are differences between dental epithelial cells and mesenchymal cells, there may be a shared switching mechanism from the cell proliferative state to the differentiation state in the developing tooth. Cell–cell communication proteins, such as Cx43 and Panx3, may play roles in this mechanism in ameloblasts and odontoblasts, respectively. It may be effective to control in ameloblasts, not by transcription, but by post-transcriptional miRNAs with ATP release to stop the proliferation of ameloblasts and switch to differentiation of pre-ameloblasts on the tooth surface. In fact, ameloblast cell division does not completely occur during the enamel layer structure formation; it is formed one layer a day from the inside to the outside of the tooth. The present results report an increase in the amount of ATP release from dental epithelial cells with an miR-1 knockdown. Five groups of channels have been identified as ATP release channels, such as connexin hemichannels, pannexin 1, calcium homeostasis modulator 1 (CALHM1), volume-regulated anion channels (VRACs), and maxi-anion channels (MACs) (Liu et al., 2008; Kinnamon and Finger, 2013; Mikolajewicz et al., 2018). Furthermore, the classical exocytosis and non-vesicular mechanisms of cellular ATP release have been reported in various cell types. Studies on the route of ATP release from miR-1 knockdown-dental epithelial cells are currently underway. The present results show that the increase in ATP release in miR-1 knockdown-dental epithelial cells was 18α-GA, a global connexin inhibitor (data not shown) (Yamada et al., 2016). Thus, an increase in extracellular ATP release in miR-1 knockdown-dental epithelial cells may be required to activate Cx43 because *Gja-1*(Cx43) is the only target gene of miR-1 in the genes encoding connexin proteins. However, the role of miR-1 in the regulation of gap junctions or hemichannel Cx43 activity, Cx43 cellular localization, or calcium oscillations in differentiating dental epithelial cells remains unknown. Further studies are required to elucidate the regulation of dental epithelial cell proliferation and differentiation by miR-1. We previously identified epiprofin as a novel member of the Sp transcription factor family expressed in certain ectodermal organs including the teeth, hair follicles, nails, skin, and limbs (Nakamura et al., 2004, 2011; Talamillo et al., 2010). The phenotype of Epfn-deficient (Epfn^–/–^) mice is partially shared with that of ODDD in humans, resulting from *Gja-1* mutations (Richardson et al., 2004; Huang et al., 2013). Our outcomes may contribute to understanding the pathogenesis of ODDD syndrome and also lead to developing novel treatments for enamel hypoplasia.

## Conclusion

We observed an inverse expression pattern between miR-1 and Cx43 in developing molars and went on to clarify their function in tooth development. During dental epithelial differentiation, the down-regulation of miR-1 induces Cx43 hemichannel formations to release intracellular ATP to the extracellular milieu, halting cell proliferation. In an enamel hypoplasia model, such as Epfn^–/–^ mice, the dysregulation of miR-1 in developing molars results in a reduction in the amount of Cx43. Although the transcriptional regulation of miR-1 in the developing tooth should be investigated, miR-1 plays important roles in enamel formation.

## Data Availability Statement

The datasets generated for this study can be found in the GEO https://www.ncbi.nlm.nih.gov/geo/query/acc.cgi?acc=GSE141608.

## Ethics Statement

The animal study was reviewed and approved by Institutional Animal Care Committee of Tohoku University.

## Author Contributions

TaN and SF designed the study and provided funding for the study. ToN, TaN, and TI participated in the entire experiment, and writing and modifying the manuscript. HN and YY helped to solve problems throughout the experiment, and participated in the editing and revision of the manuscript. TI and HN participated in the ATP release assay experiments and statistical analysis of the data. TaN, AY, KS, and YS confirmed data to prepare the revised manuscript.

## Conflict of Interest

The authors declare that the research was conducted in the absence of any commercial or financial relationships that could be construed as a potential conflict of interest.

## Supplementary Material

FIGURE S1 Results of Genopal^TM^ miRNA gene chip array in developing tooth germ.

FIGURE S2 Phase and fluorescence images after transfection of FITC-labeled scramble or miR-1 knockdown probes into SF2 cells.
