# Supplementary figures and images for: Regulation of miR-1-Mediated Connexin 43 Expression and Cell Proliferation in Dental Epithelial Cells
Source: Front Cell Dev Biol. 2020 Mar 17;8:156. doi: 10.3389/fcell.2020.00156 (PMC7089876; doi:10.3389/fcell.2020.00156)

Supplemental Fig. 1

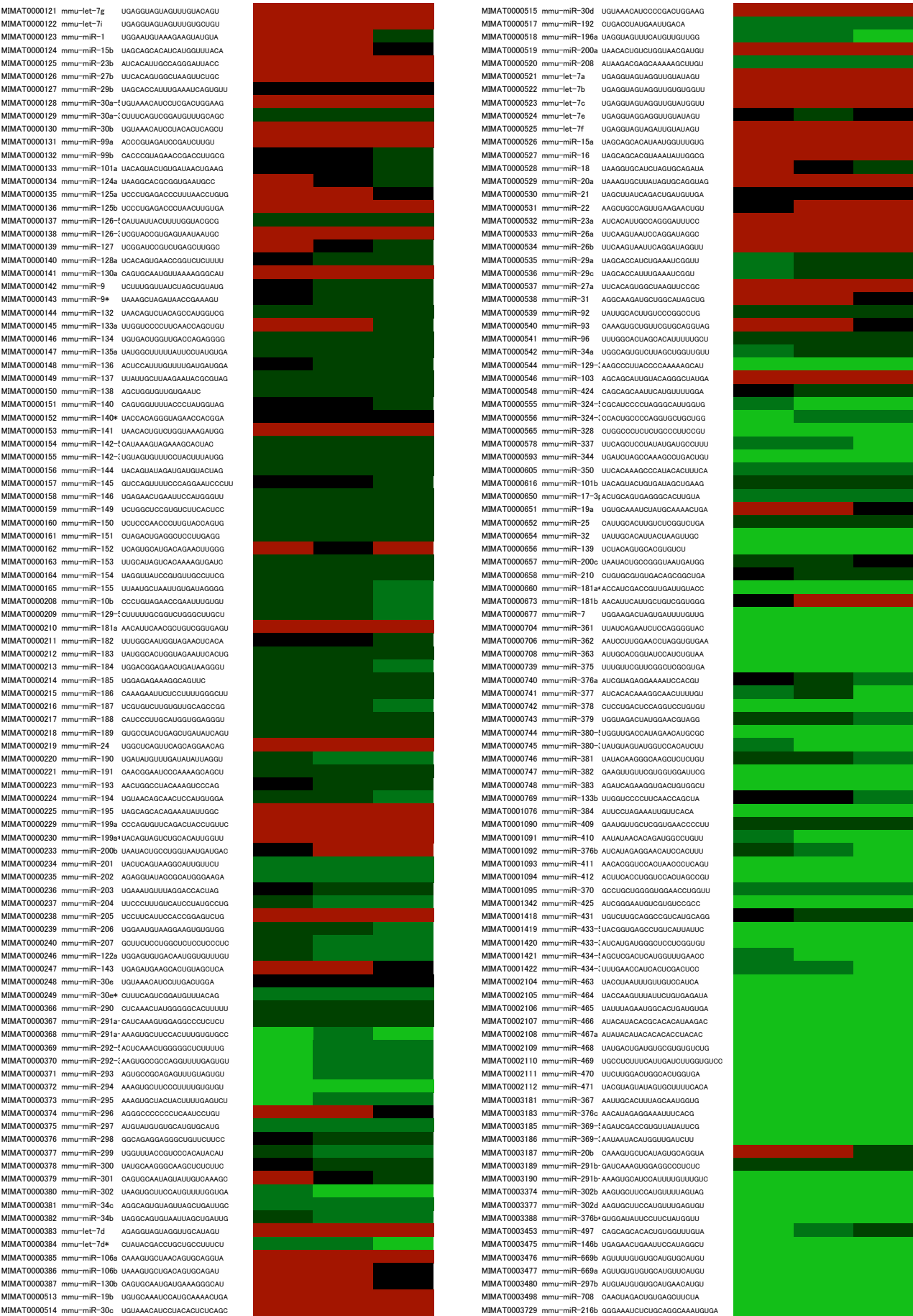

Supplement: FIGURE S1 — Results of GenopalTM miRNA gene chip array in developing tooth germ. [file Image_1.pdf]

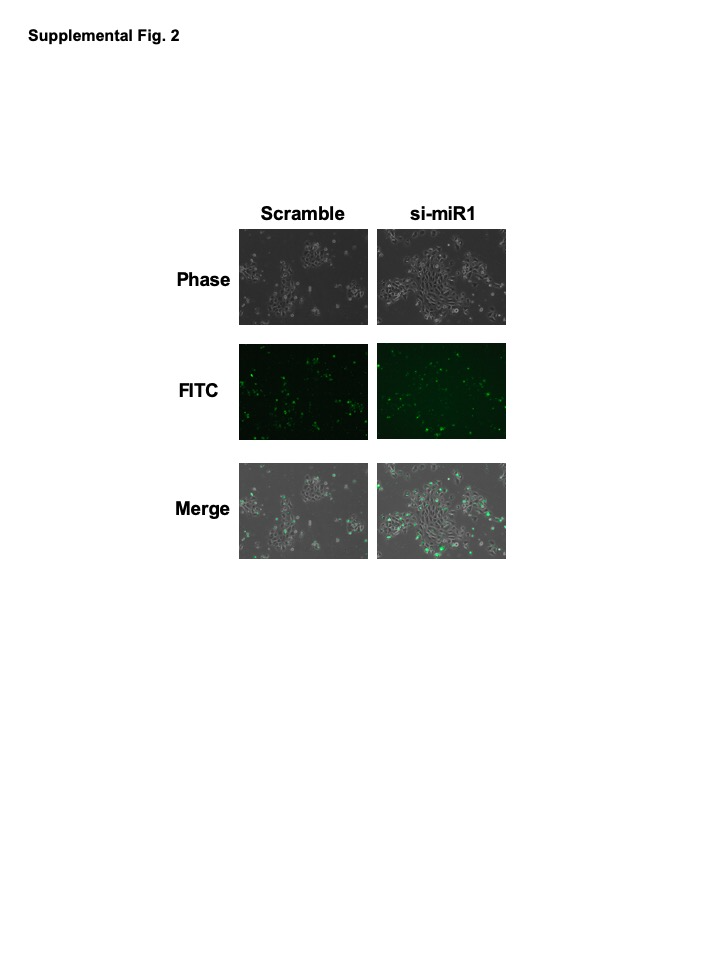

Supplement: FIGURE S2 — Phase and fluorescence images after transfection of FITC-labeled scramble or miR-1 knockdown probes into SF2 cells. [file Image_2.tiff]
